# Photo-thermo-optical modulation of Raman scattering from Mie-resonant silicon nanostructures

**DOI:** 10.1515/nanoph-2023-0922

**Published:** 2024-03-21

**Authors:** Mor Pal Vikram, Kentaro Nishida, Chien-Hsuan Li, Daniil Riabov, Olesiya Pashina, Yu-Lung Tang, Sergey V. Makarov, Junichi Takahara, Mihail I. Petrov, Shi-Wei Chu

**Affiliations:** Department of Physics, 33561 National Taiwan University, 1, Sec 4, Roosevelt Rd., Taipei 10617, Taiwan; School of Physics and Engineering, 65071 ITMO University, Lomonosova 9, Saint Petersburg 191002, Russia; Qingdao Innovation and Development Center, Harbin Engineering University, Qingdao 266000, Shandong, China; Graduate School of Engineering, Osaka University, 2-1 Yamadaoka, Suita, Osaka 565-0871, Japan; Photonics Center, Graduate School of Engineering, Osaka University, 2-1 Yamadaoka, Suita, Osaka 565-0871, Japan; Molecular Imaging Center, 33561 National Taiwan University, 1, Sec 4, Roosevelt Rd., 10617, Taipei, Taiwan; Brain Research Center, National Tsing Hua University, 101, Sec. 2, Kuang-Fu Rd., Hsinchu 300044, Taiwan

**Keywords:** photothermal effect, Mie resonance, silicon nanoparticles, Raman spectroscopy; Raman thermometry

## Abstract

Raman scattering is sensitive to local temperature and thus offers a convenient tool for non-contact and non-destructive optical thermometry at the nanoscale. In turn, all-dielectric nanostructures, such as silicon particles, exhibit strongly enhanced photothermal heating due to Mie resonances, which leads to the strong modulation of elastic Rayleigh scattering intensity through subsequent thermo-optical effects. However, the influence of the complex photo-thermo-optical effect on inelastic Raman scattering has yet to be explored for resonant dielectric nanostructures. In this work, we experimentally demonstrate that the strong photo-thermo-optical interaction results in the nonlinear dependence of the Raman scattering signal intensity from a crystalline silicon nanoparticle via the thermal reconfiguration of the resonant response. Our results reveal a crucial role of the Mie resonance spectral sensitivity to temperature, which modifies not only the conversion of the incident light into heat but also Raman scattering efficiency. The developed comprehensive model provides the mechanism for thermal modulation of Raman scattering, shedding light on the photon–phonon interaction physics of resonant material, which is essential for the validation of Raman nanothermometry in resonant silicon structures under a strong laser field.

## 1 Introduction

Silicon is widely used as the basic material of electronic and photonic devices by taking advantage of its natural abundance and well-established manufacturing process. With the progress of silicon applications, the development of efficient temperature measurement techniques becomes crucial to examine the device performance and stability [1], [2]. Among several existing thermometry techniques [3], [4], Raman thermometry [5], [6] is one of the most powerful techniques to measure the temperature of silicon materials. The monocrystalline silicon exhibits a strong and sharp Raman peak originating from the interaction of light with the optical phonon (i.e., lattice vibration), and the parameters of the Raman spectrum, such as the peak intensity, wavenumber, and linewidth, exhibit remarkable thermo-sensitivity [7]–[11]. Therefore, with proper calibration, measurement of the Raman spectrum from silicon allows one to monitor its temperature in a non-contact and non-destructive way [12]–[14], featuring an attractive advantage for industrial applications.

However, when the dimension of silicon is reduced to optical wavelength size, Raman thermometry becomes a challenging task because the optical resonance effect varies Raman scattering response so that it does not fit the conventional physical model of bulk silicon [15], [16], [17], [18], [19]. The recent discovery shows that the laser excitation of silicon nanostructures at the Mie resonance wavelength causes iterative photothermal and thermo-optical effects (termed as photo-thermo-optical effect) and results in a strong modulation of Rayleigh scattering intensity by the thermal spectral shift of resonance spectrum [20], [21], [22], [23], [24]. Previous studies found that the temperature of silicon nanoparticles obtained by Raman thermometry shows a large discrepancy from the simulation result when the Rayleigh scattering exhibits a strong modulation by the photo-thermo-optical effect [25], implying that the photo-thermo-optical effect possibly involves not only the elastic Rayleigh scattering but also the inelastic Raman scattering and causes the inaccuracy of the temperature calibration. Nevertheless, the direct measurements on the thermal modulation effect of Raman scattering intensity, especially the dependencies of Raman scattering intensity on the excitation intensity and temperature, as well as the feasibility of the Raman thermometry by using the nonlinearly modulated Raman intensity, have all not been attempted yet in the previous studies. Indeed, since there are applications requiring to irradiate a silicon nanostructure with high-power laser excitation such that overheating of structures cannot be avoided, for example, laser ablation color printing [26], optothermal reshaping of nanostructures [13], [27], [28], and optically induced phase transitions [29]. Therefore, to check the feasibility of temperature measurement of silicon nanostructure at high temperatures is highly desirable.

In this paper, we reveal a key role of the photo-thermo-optical modulation in the Raman nanothermometry for optically resonant silicon nanostructures, underlying the critical influence of the spectral dependence of Mie resonances on local temperature. We experimentally confirm the thermal modulation of Raman scattering response in crystalline silicon nanoscatterers by investigating the relationship between excitation intensity and Raman scattering spectrum in a Raman microspectroscopy. We also monitored the temperature of the nanoscatterers with two different approaches: namely Stokes and anti-Stokes (S–aS) Raman intensities ratio *I*_S_/*I*_aS_ and Stokes line shift. While Raman response in silicon has strong temperature dependence, as reported previously, we show that the photothermal shift of resonances additionally modifies the temperature measurement. The possible mechanism implies both changing the excitation conditions and Raman emission conditions due to the Purcell effect [16], [17], which may have a strong influence on Raman thermometry. As a result, a significant discrepancy between the two methods of Raman thermometry appears, and we argue that the S–aS ratio measurements are no longer reliable at high temperature. Our research provides new insight into the physics of Raman thermometry for resonant silicon nanostructures under high illumination intensity.

**Figure 1:**
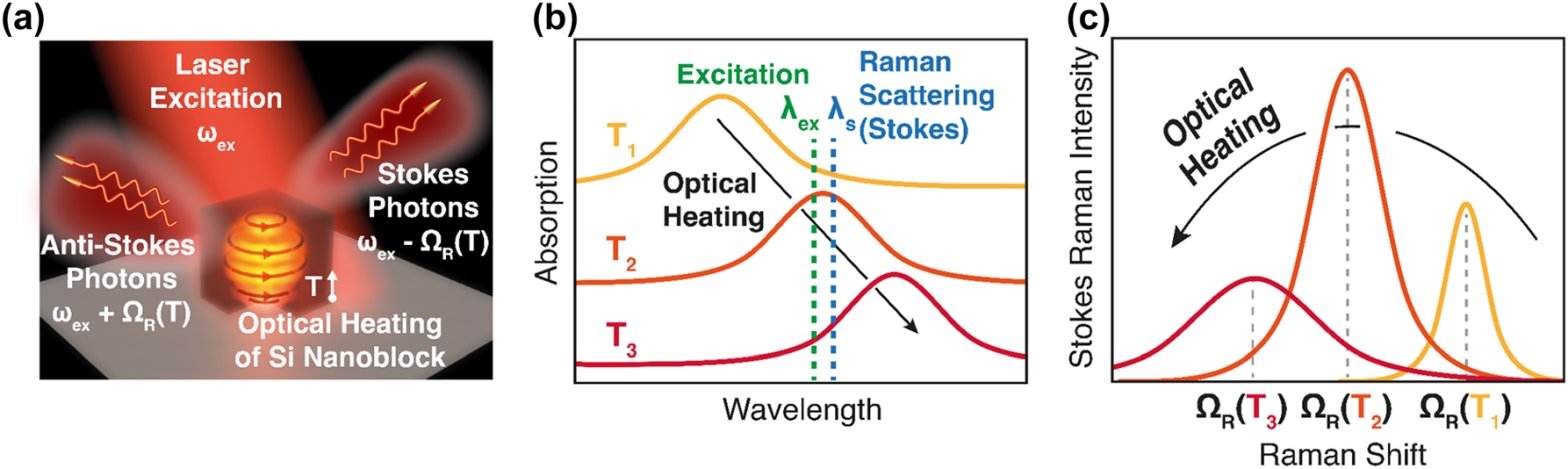
Concept for thermal modulation of Raman scattering. (a) Illustration showing that silicon nanoblock is excited by a laser beam at the frequency of *ω*_ex_ and produces Stokes (*ω*_ex_ − Ω_R_, where Ω_R_ is Raman shift) and anti-Stokes (*ω*_ex_ + Ω_R_) Raman scatterings, while the temperature of silicon nanoparticle (*T*) is increasing by the photothermal heating effect. (b) Schematics of absorption spectra at various temperatures of nanoblock (*T*_1_ (yellow) < *T*_2_ (orange) < *T*_3_ (red)), induced by optical heating (black arrow). Green and blue dotted lines indicate, respectively, the wavelengths of the excitation beam (*λ*_ex_) and Stokes–Raman scattering (*λ*_s_). (c) Schematics of temperature-dependent Stokes–Raman shift spectra corresponding to (b).

The brief principle on thermal modulation of Raman scattering from a resonant silicon nanostructure is described in Figure 1. The schematic of Figure 1(a) shows the generations of Stokes (*ω*_ex_ − Ω_R_) and anti-Stokes (*ω*_ex_ + Ω_R_) Raman scatterings, which are detuned for the Raman shift Ω_R_ from the excitation frequency *ω*_ex_, from a silicon nano-resonator with a cubic shape (silicon nanoblock) under laser irradiation, while the temperature of silicon nanoparticle (*T*) is increasing by the photothermal heating effect. Figure 1(b) and (c) are, respectively, the drawings of the temperature-dependent absorption and Stokes–Raman scattering spectrum of silicon nanoblock. Here, we assume that the absorption peak of the nanoparticle is located on the blue-side of the excitation wavelength (*λ*_ex_) at the initial low temperature state (*T*_1_), as shown in Figure 1(b). Because of this off-resonant condition, the Raman scattering signal is relatively weak at *T*_1_ (Figure 1(c)). With the growth of the excitation intensity, the temperature of the nanoparticle increases (*T*_1_ → *T*_2_), and the Mie resonant absorption peak exhibits red-shift due to the thermo-optical effect [30], resulting in the overlap with *λ*_ex_ as well as Stokes–Raman scattering wavelength (*λ*_s_). In this on-resonance condition, both excitation and emission of Raman scattering photons are efficiently enhanced by the Purcell effect [17], [31], [32]. However, further increase of the excitation intensity (*T*_2_ → *T*_3_) does not lead to more Raman signal strength but to its suppression because the absorption peak moves out of resonance with *λ*_ex_ and *λ*_s_ by stronger thermo-optical effect. Based on the above overall process, the Raman emission intensity from the nanoparticle exhibits a nonlinear relationship with excitation intensity, reflecting the complex nature of the photothermal response with subwavelength nanostructures.

In addition to the Raman intensity, the Raman shift also varies with temperature (Ω_R_(*T*)), as expressed in Figure 1(c), due to the thermal expansion of the crystal lattice and resulting phonon frequency changes [33]. On the contrary, with the nonlinear response of Raman intensity, the Ω_R_ constantly decreases with the excitation intensity because the phonon frequency is less affected by the resonance condition but is dependent only on the temperature. This property of Raman frequency makes it a more reliable parameter to calculate the temperature of the nanoparticle compared with Raman intensity, especially at high excitation intensity regimes, as discussed in the latter part of this paper. Below, we will address the mechanism for thermal modulation of Raman scattering in more detail.

## 2 Theoretical formulation

We start with the electromagnetic contribution to Raman signal enhancement and suppression, which can be interpreted within a simple coupled mode theory. In the single mode regime, the amplitude $\tilde{a}$ of the field inside the resonator is given by the coupled mode theory [17], [34].

(1) $$\frac{\text{d}\tilde{a}}{\text{d}t}=\left(-\text{i}\omega_{0}+\gamma \right)\tilde{a}+\text{i}\sqrt{\gamma_{f}}\tilde{f},$$

where *ω*_0_ is the eigenfrequency of the resonator, *γ* is the total loss coefficient, $\tilde{f}$ is the amplitude of the incident wave, and *γ_f_* is the coupling constant. The thermo-optical effect accounted for in the first order of perturbation theory through the thermo-refractive coefficient leading to the shift of the eigen frequency spectral position *ω*_0_ → *ω*_0_ − *α*| $\tilde{a}$ |^2^, where *α* is a thermo-shift coefficient. In the stationary regime under harmonic excitation $\tilde{f}$ = *f*exp(−i*ωt*), the spectral amplitude intensity of the field $\tilde{a}$ = *a*exp(−i*ωt*). Eq. (1) is immediately resolved to provide a nonlinear equation for the mode intensity [35].

At the same time, the intensity of Raman emission is governed not only by the excitation intensity but also by the density or resonant states at the Stokes frequency *ω*_s_ = *ω*_ex_ − Ω_R_ of the emission, where Ω_R_ is the phonon frequency. The enhancement of the emission due to the Purcell effect can be accounted for as follows (for more details, see Section 1 analytical model in Supplementary Materials):

(2) $$I_{\text{R}}/I_{\text{R}0}=\frac{3\Pi }{k^{3}V}\frac{\omega_{\text{ex}}\gamma }{{\left(\Delta \omega -\Omega_{\text{R}}+\alpha {\left|a\right|}^{2}\right)}^{2}+\gamma^{2}}$$

where *I*_R_ and *I*_R0_ are the Raman emission intensities with and without the account of the optical density of states behavior at the Stokes frequency, *k* is the wavevector, Ω_R_ is the Stokes frequency, and *V* is the effective volume of the mode acting in the Purcell enhancement of Raman emission, and *α* is thermoshift coefficients. One should note that the effects related to resonance at the excitation frequency correspond to the *I*_R0_ term, while Eq. (2) defines the contribution of resonant effects at the emission frequency. These models provide a qualitative description of the photo-thermo-optical effect and Purcell enhancement on Raman scattering.

## 3 Experimental results

### 3.1 Measurement of thermal modulation of Raman scattering

**Figure 2:**
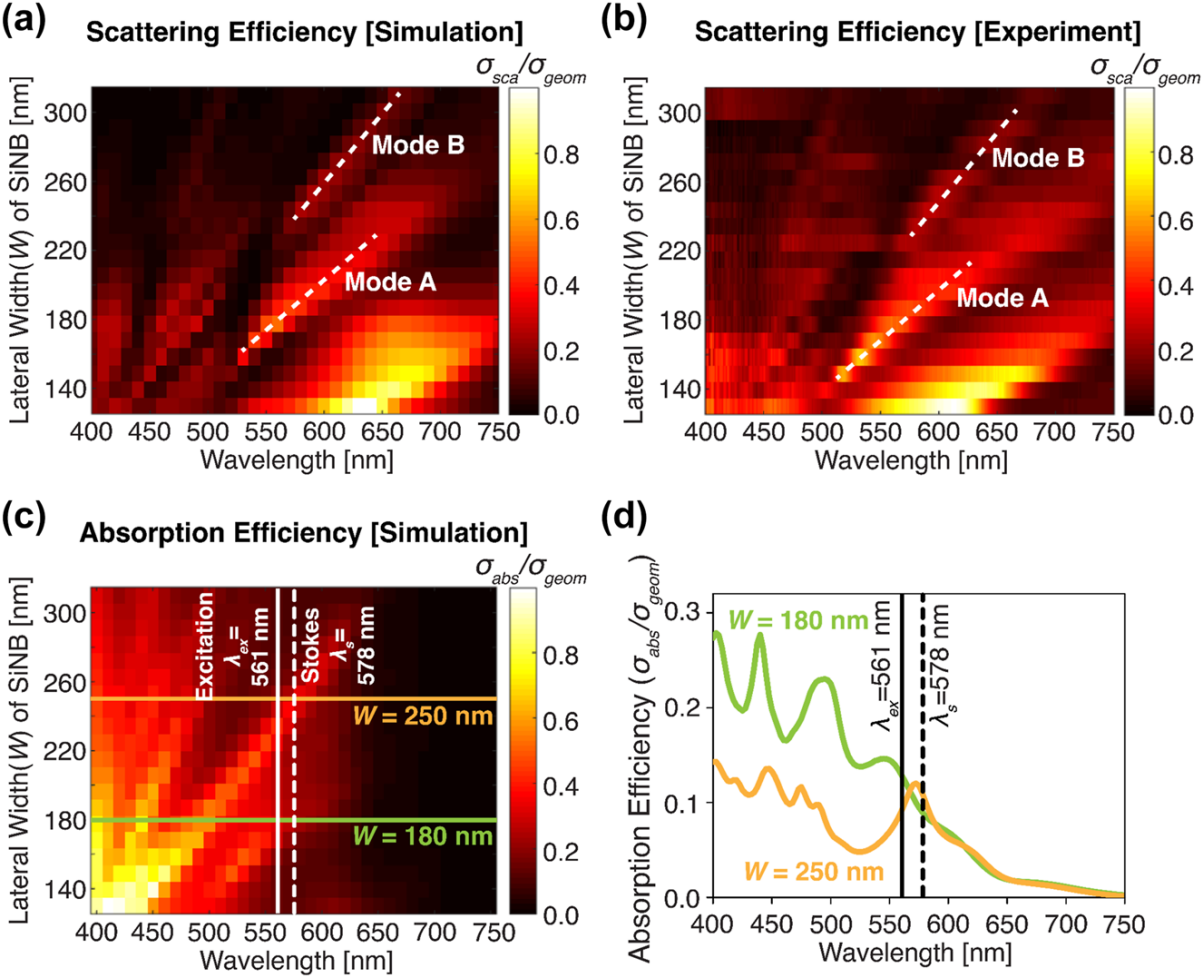
Scattering and absorption properties of silicon nanoblocks. (a, b) Scattering maps for the wavelength range from 400 to 750 nm and lateral width of silicon nanoblock from 130 to 320 nm, obtained by simulation (a) and experimental measurement (b). The height of nanoblocks is fixed at 150 nm. The nanoblocks are illuminated with p-polarized white-light under 67° incidence, and a backward scattering signal is detected as a dark-field signal collection method. The dash white lines indicate the locations of resonance: mode A and mode B. The surrounding medium of the silicon nanoblock is air. (c) Simulated absorption maps for the wavelength range from 400 to 750 nm and lateral width of silicon nanoblock from 130 to 320 nm. Solid and dashed white lines show the excitation laser wavelength (*λ*_ex_ = 561 nm) and Stokes–Raman wavelength of crystalline silicon (*λ*_s_ = 578 nm). (d) Absorption spectra of *W* = 180 nm (green solid line) and *W* = 250 nm (orange solid line) nanoblock.

Toward experimental observation of thermal modulation of Raman scattering from a silicon nanoblock, we fabricated a set of standalone crystalline silicon nanoblocks with lateral widths (*W* = *W*_*x*_ = *W*_*y*_) varied from 130 nm to 320 nm by 10 nm steps and a constant height of 150 nm (for more details of sample preparation, see Section 2 in Supplementary Materials). Figure 2(a) is the dependence of scattering spectra on the nanoblock lateral width *W*, calculated by using the commercial finite element method (COMSOL Multiphysics, COMSOL Inc.). In this scattering map, two representative Mie resonant modes, namely “Mode A” and “Mode B”, appear with controllable resonance peak wavelengths depending on *W* (the field distributions in these resonance modes are shown in Supplementary Materials Figure S1). In Figure 2(b), we experimentally confirmed Mie resonances in the fabricated silicon nanoblocks by dark-field microspectroscopy, where the individual silicon nanoblocks were illuminated with white light, and the backward scattering was collected by the objective within a dark-field design, and then sent to a spectrometer (see Figure S3 in Section 3 of Supplementary Materials). The experimental results show excellent correspondence to the calculated result (Figure 2(a)), manifesting high-quality fabrication.

We also calculated the absorption map of silicon nanoblock alongside the excitation laser wavelength (*λ*_ex_ = 561 nm) and Raman–Stokes wavelength of crystalline silicon (*λ*_s_ = 578 nm) in Figure 2(c), and specifically showed the individual absorption spectra of *W* = 180 nm and 250 nm silicon nanoblocks in Figure 2(d). Note that we assumed that the silicon nanoblock is fixed on the quartz substrate and the surrounding medium is objective immersion oil in this simulation, in order to match the condition with our Rayleigh and Raman scattering measurements shown in the next section, which required the use of a high NA (=1.40) oil immersion objective lens. In Figure 2(d), we confirmed that the resonant absorption peak of *W* = 180 nm silicon nanoblock is located at the blue-side of both the excitation wavelength and Stokes–Raman scattering wavelength, which agree with the situation of Figure 1 and is expected the large Raman scattering modulation by photo-thermo-optical effect. On the other hand, the absorption peak of the *W* = 250 nm silicon nanoblock locates the red-side of the excitation wavelength and thus should not induce strong modulation.

To experimentally confirm our theory on the thermal modulation of scattering signals, we experimentally measured both the Rayleigh and Raman scattering spectra for two representative sizes of silicon nanoblock: *W* = 180 nm that does fulfill the condition or resonance wavelength for large thermal modulation, and *W* = 250 nm that does not, as discussed in Figure 2(d). We used single-wavelength laser irradiation by using a confocal laser scanning microscope equipped with a spectrometer (detailed optical setup is shown in Section 3 of Supplementary Materials). In the experiment, we placed a tightly focused laser spot by the objective lens with an NA of 1.40 onto a single silicon nanoparticle and detected the scattering spectrum, while varying the excitation intensity. The excitation light source is a continuous-wave (CW) laser oscillating at the wavelength of 561 nm.

**Figure 3:**
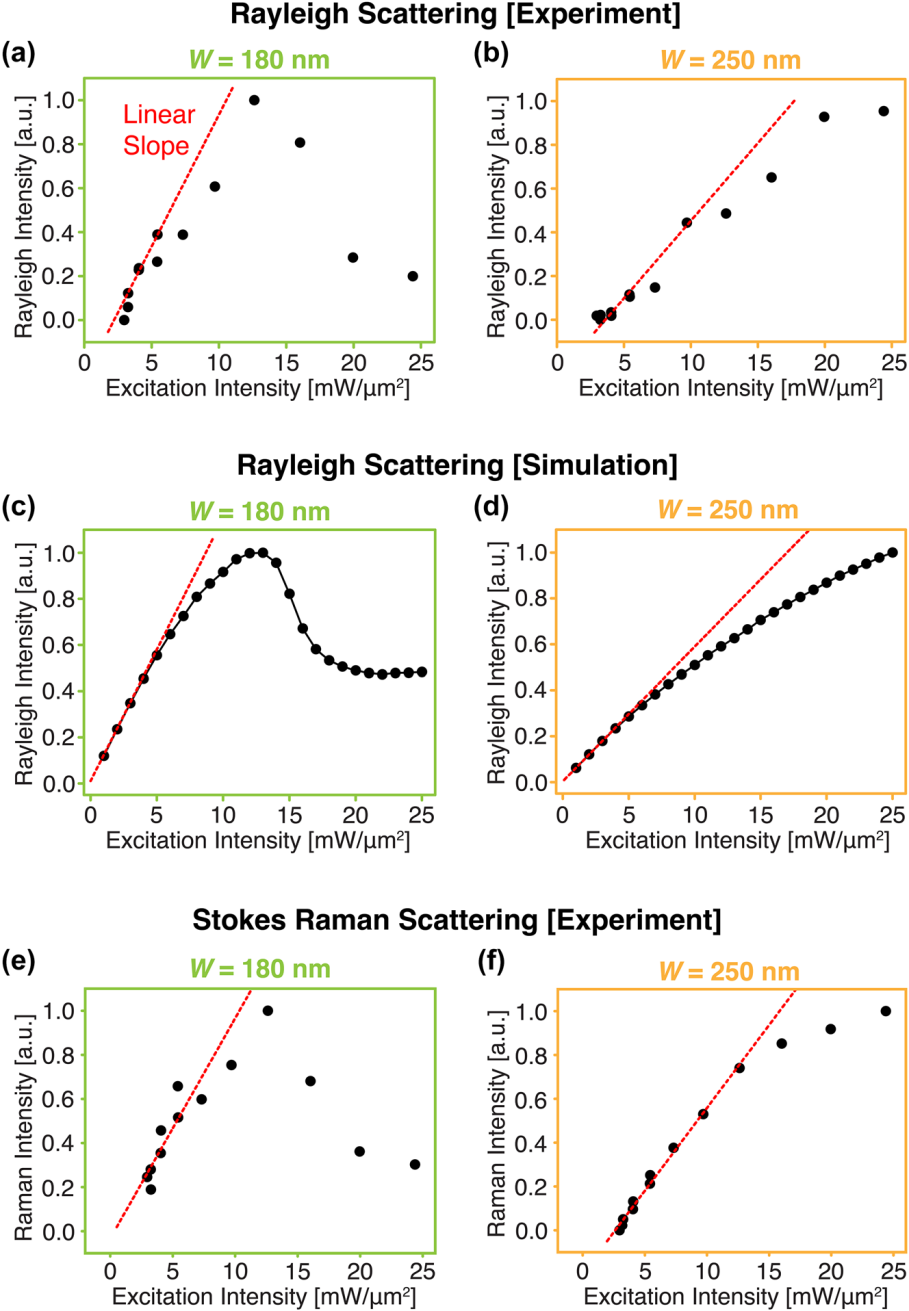
Dependences of Rayleigh and Raman scatterings from single silicon nanoblocks (*W* = 180 nm and 250 nm) on excitation intensity. (a, b) Experimentally measured relationships between Rayleigh scattering intensity of *W* = 180 nm (a) and 250 nm (b) silicon nanoblock and excitation intensity. (c, d) Calculated relationships between Rayleigh scattering intensity of *W* = 180 nm (c) and 250 nm (d) silicon nanoblock and excitation intensity. (e, f) Experimentally measured relationships between Raman scattering intensity of *W* = 180 nm (e) and 250 nm (f) silicon nanoblock and excitation intensity.

Figure 3(a) and (b) are the experimentally measured relationships between excitation and Rayleigh scattering intensities from *W* = 180 nm and 250 nm silicon nanoblocks, respectively. Under low laser excitation intensity, the Rayleigh scattering intensities of both nanoblocks basically show proportional increase with the excitation intensity. However, by further increasing the laser illumination, the Rayleigh scattering intensities start to deviate from linear trend because the photo-thermo-optical effect modulates the scattering cross-sections of silicon nanoblocks. The *W* = 180 nm nanoblock (Figure 3(a)) represents a significant decrease of scattering intensity (i.e. sub-linear) after the excitation intensity exceeds 12 mW/μm^2^. On the other hand, in the *W* = 250 nm nanoblock (Figure 3(b)), although the sub-linear trend of scattering intensity is observed as the excitation intensity becomes higher, the deviation from linear trend is not as dramatic as that in the *W* = 180 nm nanoblock. This is because the resonance wavelength of the *W* = 250 nm nanoblock starts to move far from *λ*_ex_ = 561 nm with the temperature increases as discussed in Figures 1 and 2, and the induced photothermal effect becomes significantly weaker compared with that of the *W* = 180 nm nanoblock. As shown in Figure 3(c) and (d), these experimental results were confirmed by the numerical calculation using commercial finite element method software (the calculation condition is described in Section 4 of Supplementary Materials), representing good agreements with each other.

In Figure 3(e) and (f), we plot the dependencies of Stokes–Raman scattering peak intensities from the *W* = 180 nm and 250 nm silicon nanoblocks versus excitation intensity. The Raman scattering intensities from both the *W* = 180 nm and 250 nm nanoblocks exhibit obvious nonlinearly responses to the excitation intensity. Because bulk silicon does not show nonlinear response of Raman scattering intensity (see Figures S5 and S6 in Section 5 of Supplementary Materials), the origin of the Raman modulation strongly relates to the Mie resonance of nanostructure. Moreover, the overall trends of both Raman scattering curves well correspond with those of the Rayleigh scattering ones (Figure 3(a) and (b)). These facts indicate the photo-thermo-optical effect of silicon Mie resonator causes modulation of Raman scattering. It should be noted that the wave numbers of Raman shift became lower as the excitation intensity increased, indicating the phonon frequency variation due to the thermal expansion of crystalline silicon (full Raman spectrum and Raman shifts of silicon nanoblocks at various excitation intensities are shown in Figures S8 and S9 of Section 6, in Supplementary Materials).

### 3.2 Temperature calculation from Raman spectra

**Figure 4:**
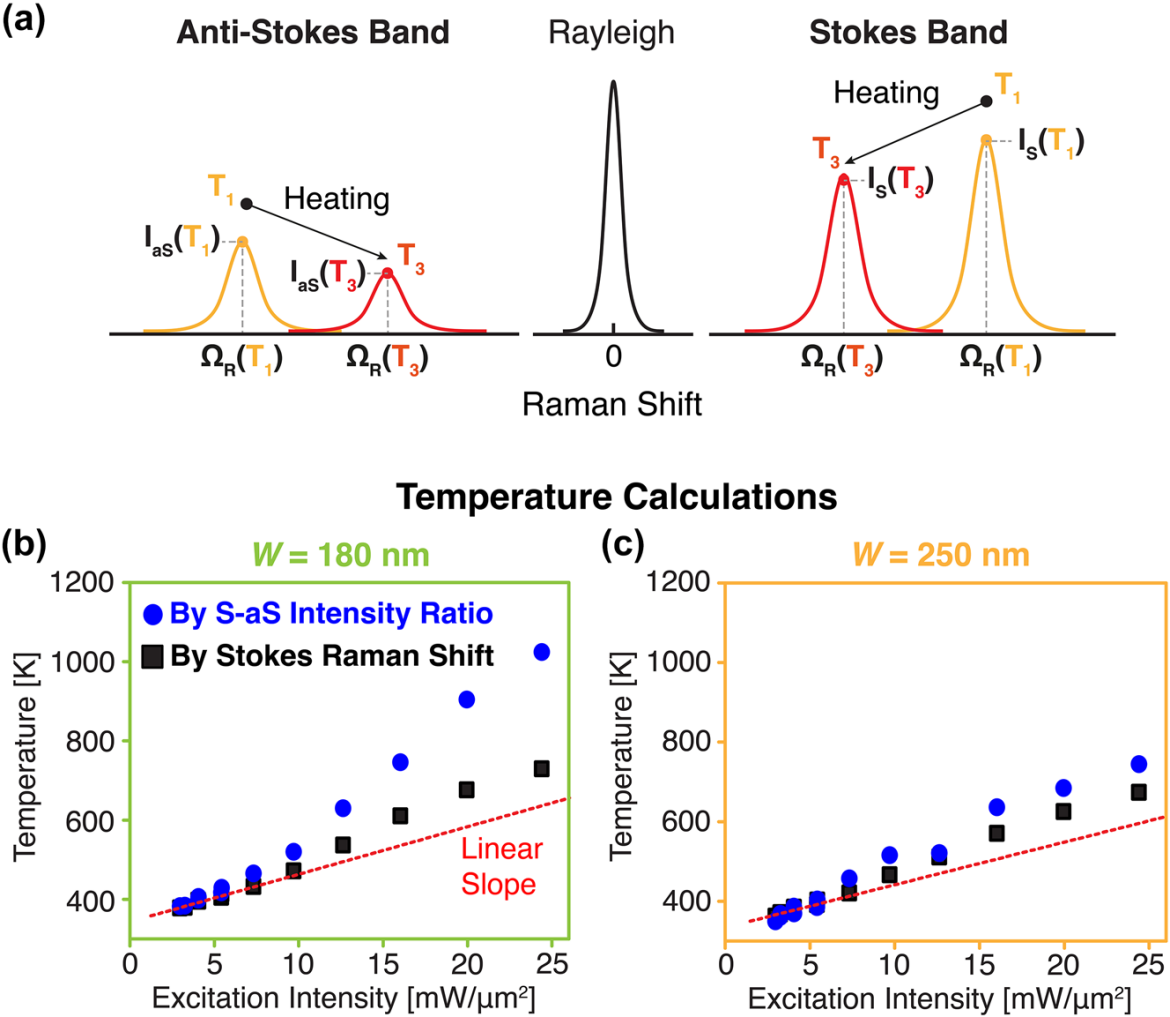
Temperature calculation from Raman spectra (a) Schematic representation of temperature-dependent Stokes and anti-Stokes spectra, where *I*_S_ and *I*_aS_ are respectively Stokes and anti-Stokes peak intensities, Ω_R_ is the wavenumber of Raman shift, and *T* is the temperature (*T*_3_ >> *T*_1_). (b, c) Temperatures of silicon nanoparticles calculated based on the Stokes and anti-Stokes–Raman scattering peak intensity ratio (blue circle) and Stokes peak wavenumber (black rectangle) for *W* = 180 nm (b) and 250 nm (c) silicon nanoblock, respectively.

We attempted the calculation of silicon nanoblock temperatures by analyzing the Raman spectra obtained in the experiment, in order to discuss the feasibility of Raman thermometry in the regime where Raman intensity shows the thermal modulation. As shown in schematic Figure 4(a), the parameters of the Stokes and anti-Stokes spectra, especially the peak intensity (*I*_S_ and *I*_aS_) and Raman shift (Ω_R_), exhibit remarkable temperature dependences. We derived the absolute temperature of the silicon nanoblock by using two different approaches. The first approach is via the peak intensity ratio of Stokes versus anti-Stokes (S–aS), which shall be significantly affected by the photo-thermo-optical effect in Mie resonance through the Purcell effect, namely an increase in the optical density of states. Another approach is based on the wavenumber of Stokes–Raman shift. The absolute peak wavenumber is less affected by the photo-thermo-optical effect because it basically depends only on the phonon frequency of the sample. Therefore, the comparison of the temperatures obtained by these two thermometry approaches allows us to examine the contribution of the photo-thermo-optical modulation effect in the Raman scattering spectra to Raman thermometry. The theoretical models used in these two temperature calculations are described in Section 7 of Supplementary Materials. Note that we confirmed these Raman thermometry methods properly worked to measure the temperature of bulk silicon without significant discrepancy between two approaches (see Figure S7 in Section 5 of Supplementary Materials).

In Figure 4(b), we show the experimentally estimated temperature based on the S–aS intensity ratio and the peak position of Raman scattering from the *W* = 180 nm silicon nanoblocks. The peak intensity and the peak position were determined by applying the Lorentzian curve fitting to the experimental Raman spectra. The temperature calculated by intensity ratio exhibits a sharp super-linear increase in the excitation intensity and finally reaches over 1020 K at the excitation intensity of 25 mW/μm^2^. However, the temperatures calculated by the peak positions approximately follow a linear trend and reach only 730 K at the same 25 mW/μm^2^, representing about 300 K difference from the temperature of the S–aS intensity ratio. On the other hand, the temperatures of the *W* = 250 nm silicon nanoblock are shown in Figure 4(c), where the temperature derived from the intensity ratio and from peak position are much closer to each other. Specifically, the temperature deviation is only ∼15 % in *W* = 250 nm nanoblock at the excitation intensity of 25 mW/μm^2^, while the temperature deviation is 100 % in *W* = 180 nm nanoblock. This fact implies that the Raman peak intensity is largely affected by the photo-thermo-optical modulation effect and the Purcell effect, which is induced by Mie resonance in *W* = 180 nm silicon nanoblock, resulting in inaccurate temperature estimation via the intensity ratio method. Therefore, peak wavelength shift is a better parameter for Raman thermometry, compared with Raman peak intensity, under high excitation intensity that the Raman scattering exhibits thermal modulation.

## 4 Discussion

We demonstrate that Raman scattering intensity from Mie-resonant silicon nanostructures exhibits nonlinear variation versus the excitation intensity via the photo-thermo-optical modulation effect. The thermally induced nonlinear trend is verified by the concurrent Rayleigh scattering nonlinear behavior. To identify the impact of photo-thermo-optical modulation on temperature measurement, we compared Raman thermometry based on the S and aS intensity ratio and on the Raman shift peak position. A significant discrepancy is found for the *W* = 180 nm silicon nanoblock, while for the *W* = 250 nm silicon nanoblock, the two thermometry results agree better, implying the significant contribution of the Purcell effect in the modulation of Raman scattering. Previously, many research groups attempted Raman nanothermometry, but they did not find large temperature discrepancies between the two methods based on S–aS ratio and Stokes wavenumber because they focused on the linear Raman scattering intensity region [12], [21]. To use Raman peak intensity for thermometry of resonant nanostructure in this thermally modulated scattering regime, further calibration based on the physical model considering photo-thermo-optical effect is required. Our study offers a new insight into the photon–phonon interaction physics at the nanoscale, which is essential to validate Raman thermometry under a strong excitation field.

One important question is the relative contribution of the field enhancement at the excitation wavelength and the Purcell effect at the emission wavelength on the observed Raman scattering modulation. Although experimentally to disentangle these two effects is difficult, theoretically we are able to estimate it based on the absorption spectrum of the silicon nanoblock. As shown in Figure 2(d), the *W* = 180 nm silicon nanoblock exhibits the absorption peak (i.e. Mie resonance peak) onto the excitation wavelength, while it does not correspond with the Stokes–Raman wavelengths. Therefore, in this case, the contribution of the field enhancement effect at the excitation wavelength might become more important than the Purcell effect at the emission wavelength. On the other hand, the *W* = 250 nm nanoblock exhibits the Mie resonance at the Stokes–Raman wavelength, making the contribution of the Purcell effect significant. In order to verify the contribution of the Purcell effect, we performed the numerical simulation of Raman scattering intensity considering with and without the Purcell effect, for the *W* = 180 nm and *W* = 250 nm silicon nanoblocks, by using commercial finite element method (FEM) software as shown in Section 8 of Supporting Information. Our numerical calculation indicates that the *W* = 250 nm silicon nanoblock Raman intensity shows a larger difference between with and without the Purcell effect than that of the *W* = 180 nm nanoblock. The result confirms the more significant contribution of the Purcell effect when the resonant peak coincides with the Raman emission band.

## Supplementary Material

Supplementary Material Details
