# Supplementary material for: Photo-thermo-optical modulation of Raman scattering from Mie-resonant silicon nanostructures
Source: Nanophotonics. 2024 Mar 21;13(18):3581–9. doi: 10.1515/nanoph-2023-0922 (PMC11501727; doi:10.1515/nanoph-2023-0922)
Supplement: Supplementary file 1 — Supplementary Material Details [file j_nanoph-2023-0922_suppl_001.pdf]

# Supplementary Materials:

# Photo-thermo-optical modulation of Raman scattering from Mie-resonant silicon nanostructures

Mor Pal Vikram[1], Kentaro Nishida[1]*, Chien-Hsuan Li[1], Daniil Riabov[2], Olesiya Pashina[2], Yu-Lung Tang[1], Sergey V. Makarov[2,3], Junichi Takahara[4,5], Mihail I. Petrov[2]*, and Shi-Wei Chu[1,6,7]*

[1]Department of Physics, National Taiwan University, 1, Sec 4, Roosevelt Rd., Taipei 10617, Taiwan

[2]School of Physics and Engineering, ITMO University, Lomonosova 9, Saint Petersburg 191002, Russia

[3]Qingdao Innovation and Development Center, Harbin Engineering University, Qingdao 266000, Shan-dong, China

[4]Graduate School of Engineering, Osaka University, 2-1 Yamadaoka, Suita, Osaka 565-0871, Japan

[5]Photonics Center, Graduate School of Engineering, Osaka University, 2-1 Yamadaoka, Suita, Osaka 565- 0871, Japan

[6]Molecular Imaging Center, National Taiwan University, 1, Sec 4, Roosevelt Rd., 10617, Taipei, Taiwan

[7]Brain Research Center, National Tsing Hua University, 101, Sec. 2, Kuang-Fu Rd., Hsinchu 300044, Taiwan

*Corresponding authors:

S.-W. Chu swchu@phys.ntu.edu.tw,

M. Petrov trisha.petrov@gmail.com,

K. Nishida knishida@phys.ntu.edu.tw

## • Section 1. Analytical Model

We start from the model of a single mode resonator, in which the amplitude "*a*" an equation could be written as[1]:

$$\frac{d\tilde{a}}{dt} = (-i\omega_0 + \gamma)\tilde{a} + i\sqrt{\gamma_f}\tilde{f}, \tag{1}$$

where $\omega_0$ is the eigenfrequency of the resonator; $\gamma$ is the total loss coefficient; $\tilde{f}$ is the amplitude of the incident wave (normalized as $|\tilde{f}|^2$ = power carried by the incident wave), and $\gamma_f$ corresponds to the radiation losses channel responsible for the resonator excitation. The amplitude $|a|^2$ is normalized to the energy of the optical mode. If we consider thermorefraction, eigenfrequency position $\omega_0$ and total loss coefficient $\gamma$ change and equation (1) should be then modified as:

$$\frac{d\tilde{a}}{dt} = (-i\omega_0 + i\alpha|a|^2 + \gamma + \beta|a|^2)\tilde{a} + i\sqrt{\gamma_f}\tilde{f}, \tag{2}$$

where $\alpha$ and $\beta$ are thermoshift coefficients. If we consider the incident wave in the form of $\tilde{f}$ = $f\exp(-i\omega t)$ where $\omega$ is the excitation wave frequency, then we can search the solution as $\tilde{a} = a\exp(-i\omega t)$ as well. Substituting it in the equation (2), we obtain for the amplitude:

$$a = \frac{if\sqrt{\gamma_f}}{i\Delta\omega - i\alpha|a|^2 - \gamma - \beta|a|^2}, \tag{3}$$

where $\Delta\omega = \omega_0 - \omega$ and for the amplitude squared:

$$|a|^2 = \frac{\gamma_f|f|^2}{(\gamma + \beta|a|^2)^2 + (\Delta\omega - \alpha|a|^2)^2} \tag{4}$$

So, to calculate the amplitude in a single-mode resonator being excited by the incident beam, one needs to solve the nonlinear equation (3). The electric field amplitude of the mode should be then defined as $\mathbf{E} = |a| \cdot \mathbf{M}(\mathbf{r})/\sqrt{\varepsilon_0}$, where $\mathbf{M}(\mathbf{r})$ is the vector function of excited mode field distribution (normalized as in Ref. [2]).

Raman emission signal could be considered in an incoherent way separately for three phonon polarizations. The Raman intensity of a particular phonon mode $\sigma$ is then given by[3].

$$\langle I_R^\sigma \rangle = \frac{v\omega_s^4\mu_0}{12\pi c}\int dV F_p^\sigma(\omega_s, \mathbf{r})\,|P_R^\sigma(\mathbf{r}, \omega_s, \omega)|^2, \tag{5}$$

where $v$ is the normalization volume, $\omega_S$ is the Stokes frequency, $\mu_0$ is the magnetic permittivity of vacuum, $c$ is the speed of light, $F_R^\sigma(\omega_s, \mathbf{r})$ is the Purcell factor, $P_R^\sigma(\mathbf{r}, \omega_s, \omega)$ is induced Raman polarization and the integration is taken over the nanoparticle volume.

The Purcell factor could be introduced in terms of the Green's function as[3]:

$$F_p^\sigma(\omega,\mathbf{r}) = \frac{6\pi c}{\omega}\left[\mathbf{n}_R^{\sigma *}\,\mathrm{Im}\,\widehat{\mathbf{G}}(\omega,\mathbf{r},\mathbf{r})\mathbf{n}_R^\sigma\right], \tag{6}$$

where $\mathbf{n}^\sigma{}_R$ is the unit vector of Raman polarization $\mathbf{P}^\sigma{}_R = P_R{}^\sigma\mathbf{n}^\sigma{}_R$ and $\widehat{\mathbf{G}}(\omega,\mathbf{r},\mathbf{r})$ is the dyadic Green's function of the investigated system which could be expanded into the series of resonant state vector fields products as[2]:

$$\hat{G}_k(r,r') = \sum_n \frac{Mn(r)\otimes M_n(r')}{2k(k-k_n)} \tag{7}$$

where $\mathbf{M}_n(\mathbf{r})$ is the normalized electric field distribution of the eigenmode, and $k_n$ is its wave vector which is complex in general. In the case of a single-mode optical resonator, the series of vector field products (5) is restricted by one term, and the complex wavevector should be presented as $k_0 = (\omega_0 - \alpha|a|^2 + i\gamma + i\beta|a|^2)/c$.

Raman polarization for a particular phonon polarization $\sigma$ is defined through the Raman tensor $R^\sigma$ as[3]:

$$\mathbf{P}_R^\sigma(\mathbf{r}) = \alpha_R\widehat{R}^\sigma\mathbf{E}(\mathbf{r}) = |a|\alpha_R\widehat{R}^\sigma\cdot\mathbf{M}(\mathbf{r})/\sqrt{\varepsilon_0}, \tag{8}$$

where $\alpha_R$ is the phonon-polarization independent scalar. The unit vector of the Raman polarization could be therefore expressed as:

$$\mathbf{n}_R^\sigma = \frac{\widehat{R}^\sigma\cdot\mathbf{M}(\mathbf{r})}{\left|\widehat{R}^\sigma\cdot\mathbf{M}(\mathbf{r})\right|} \tag{9}$$

Inserting this expression into equation (4) and supposing the vector field function $\mathbf{M}(\mathbf{r})$ to be real, we obtain for the Purcell factor:

$$F_p^\sigma(\omega,\mathbf{r}) = \frac{6\pi c}{\omega}\frac{1}{\left|\widehat{R}^\sigma\cdot\mathbf{M}(\mathbf{r})\right|^2}\mathrm{Im}\left\{\frac{1}{2\frac{\omega}{c}\frac{(\omega-\omega_0)+\alpha|a|^2-i(\gamma+\beta|a|^2)}{c}}\right\}\left\{\widehat{R}^\sigma\mathbf{M}(\mathbf{r})\right\}^\dagger\mathbf{M}(\mathbf{r})\otimes\mathbf{M}(\mathbf{r})\left\{\widehat{R}^\sigma\mathbf{M}(\mathbf{r})\right\} =$$

$$= \frac{3\pi}{k^3}\frac{\omega(\gamma+\beta|a|^2)}{\left\{(\Delta\omega-\alpha|a|^2)^2+(\gamma+\beta|a|^2)^2\right\}}\frac{\left|\mathbf{M}(\mathbf{r})\cdot\widehat{R}^\sigma\cdot\mathbf{M}(\mathbf{r})\right|^2}{\left|\widehat{R}^\sigma\cdot\mathbf{M}(\mathbf{r})\right|^2} \tag{10}$$

Finally, we get the expression for the particular phonon mode Raman intensity:

$$\langle I_R^\sigma \rangle = \underbrace{\frac{v\omega_s^4\mu_0}{12\pi c}\alpha_R^2\frac{|a|^2}{\varepsilon_0}}_{I_{R0}^\sigma}\int dV F_p^\sigma\left(\omega_s,\mathbf{r}\right)\left|\widehat{R}^\sigma\cdot\mathbf{M}(\mathbf{r})\right|^2 =$$

$$= I_{R0}^\sigma\frac{3\pi}{k^3V_\sigma}\frac{\omega\left(\gamma+\beta|a|^2\right)}{\left(\Delta\omega_s-\alpha|a|^2\right)^2+\left(\gamma+\beta|a|^2\right)^2} = I_{R0}^\sigma F_{p,\mathrm{eff}}^\sigma, \tag{11}$$

where $\Delta\omega_s = \omega_0 - \omega_s = \omega_0 - \omega + \Omega = \Delta\omega + \Omega$, $\Omega$ is the Stokes frequency, and $V_\sigma$ is the effective volume of the mode acting in the Purcell enhancement of Raman emission

$$\frac{1}{V_\sigma} = \int dV\left|\mathbf{M}(\mathbf{r})\cdot\widehat{R}^\sigma\cdot\mathbf{M}(\mathbf{r})\right|^2, \tag{12}$$

while $F_{p,}{}^\sigma{}_{\mathrm{eff}}$ is the Purcell factor for the particular Raman phonon mode $\sigma$ averaged over the resonator volume. As one can see, at the resonance, it acquires a form similar to a classical expression $3\pi Q/(k^3V_\sigma)$, where $Q$ is the Q-factor of the mode. Full Raman intensity is obtained by summing over the phonon polarization:

$$S_R = \sum_{\sigma=x,y,z}\langle I_R^\sigma\rangle\,. \tag{13}$$

Solving the nonlinear equation (3) on amplitude $|a|^2$ we can introduce Raman signal dependence as:

$$I_R \sim \frac{\left(\gamma+\beta|a|^2\right)}{\left(\Delta\omega_s-\alpha|a|^2\right)^2+\left(\gamma+\beta|a|^2\right)^2}\cdot|a|^2 \tag{14}$$

One can see from the formula that for large input intensities (and hence large values of amplitude $|a|^2$) Raman intensity converges to the constant value:

$$I_R \rightarrow \frac{\beta}{\alpha^2+\beta^2} \tag{15}$$

## • Section 2. Sample Preparation

In our experiment, we prepared crystalline silicon nanoblocks with variable side widths from 130 nm to 320 nm (the height is for all 150 nm). The monocrystalline silicon on the quartz substrate, which is fabricated by wafer bonding at the temperature of less than a thousand degrees after hydrogen ion implementation to the silicon wafer surface[4], was provided by Shin-Etsu Chemical Co., Ltd. For the fabrication of silicon nanoblocks, a chemical resist (SEP 520 A, Zeon Corp.) was first spin-coated on the silicon layer, and a structural pattern was drawn by electron beam lithography (ELS-7700T Elionix Inc.). After that, an electron beam evaporated a Cr layer with a thickness of 30 nm on the sample, and Cr mask patterns were formed after lifting off the resist. The silicon layer was then selectively etched by $SF_6$ and $C_4F_8$ plasma gas in a reactive ion etching chamber. Finally, the Cr mask was removed by immersing the sample in di-ammonium cerium(IV) nitrate solution[5]. Note that all fabricated silicon nanoblocks are isolated at enough distance (10 μm) in order to avoid the thermal effect or optical coupling from the neighboring nanoblock during measurement.

As shown in Figures 2(a) and 2(b) in the main manuscript, the silicon nanoblocks exhibit two representative resonance modes, namely “Mode A” and “Mode B”. The calculation of the field distribution inside the nanoblock and the multipole decomposition analysis in these resonances are performed by using the commercial finite element method (FEM) (COMSOL Multiphysics, COMSOL Inc.), as respectively shown in Figure S1 and Figure S2. According to Figure S2(a), Mode A in a W = 180 nm silicon nanoblock is influenced by different order modes, specifically the first order (magnetic and total electric dipoles) and the second order (magnetic and electric quadrupoles). On the other hand, for resonance Mode B in the W = 250 nm silicon nanoblock, the magnetic dipole primarily drives the resonance, but higher-order modes also play a significant role.

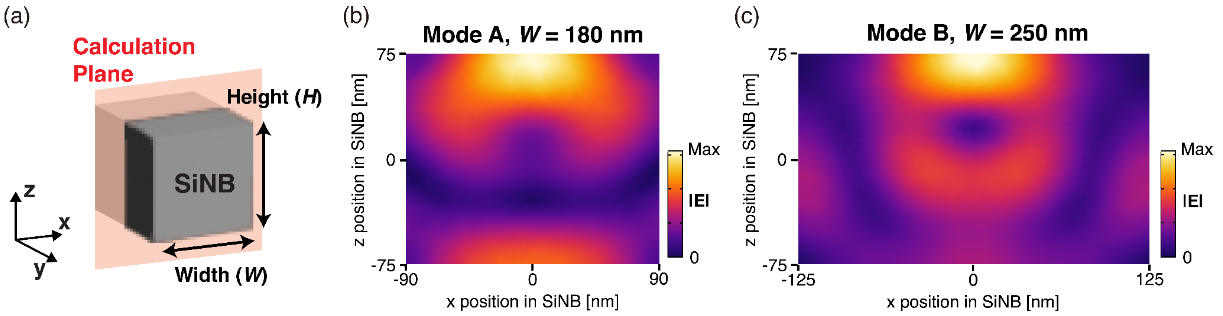


Figure S1: Calculated electric field distribution in a silicon nanoblock (SiNB). (a) Calculation setup. The calculation plane is located in the middle of the nanoblock in the y-direction. (b, c) Field distributions of Mode A in $W$= 180 nm nanoblock (b) and Mode B in $W$= 180 nm nanoblock (c). The excitation wavelength is 561 nm.

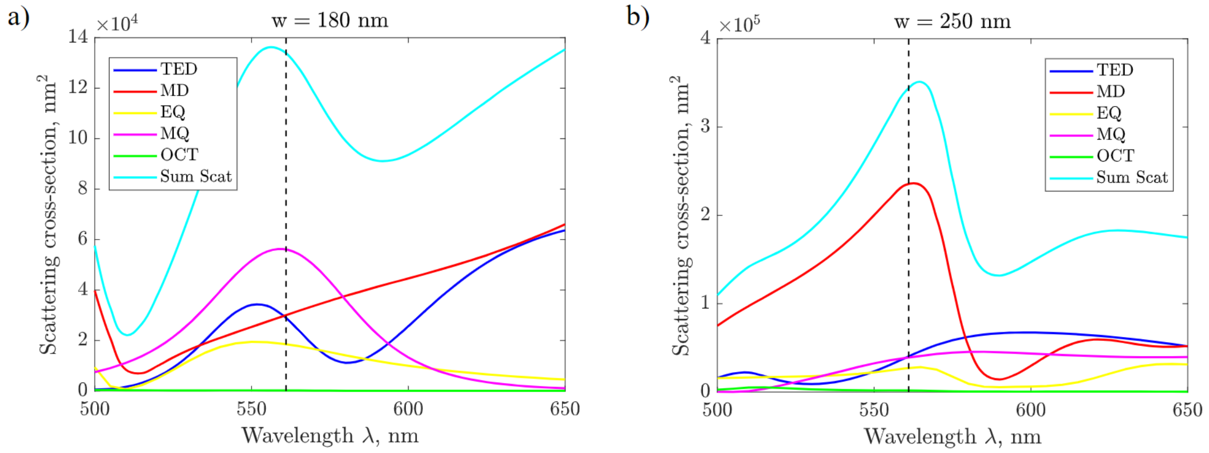


Figure S2: Spectra of the scattering cross sections and corresponding multipoles were calculated for silicon nanoblocks of different sizes: W=180 nm (a) and W=250 nm (b). The blue line represents the total electric dipole moment (TED), the red line corresponds to the magnetic dipole moment (MD), the yellow line shows the electric quadrupole moment (EQ), the purple line refers to the magnetic quadrupole moment (MQ), and the green one demonstrates the electric octupole moment (OCT). The aquamarine color is used to illustrate the summarizing scattering cross-section for the nanoparticle. The dotted line indicates the excitation wavelength of 561 nm.

## • Section 3. Experimental Setups

### White-light scattering spectrum measurements

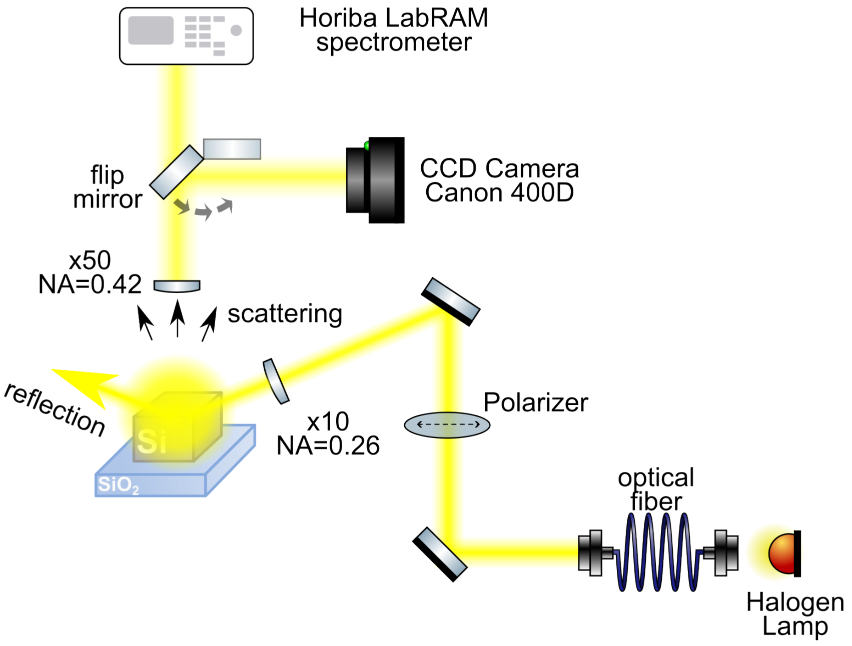


Figure S3. Dark-field spectroscopy setup for white-light scattering measurements.

In order to experimentally observe the while light scattering spectrum in the silicon nanoblocks on the quartz substrate (Figure 2(b) in the main manuscript), we perform the optical scattering measurements utilizing a dark-field spectroscopy setup. The schematic view of the approach is shown in Figure S3. The broadband light from the halogen lamp is coupled to the optical fiber and brought through the collimator. After passing through the two tunable mirrors and the polarizer, it is focused on the sample stage with an objective lens (10x, NA= 0.26, Mitutoyo Plan Apo NIR) under oblique incidence, namely 67 degrees with respect to the sample surface normal. Part of the light reflects from the substrate, while scattering signal from the silicon nanoblocks is scattered in the backward direction and further collected by the objective (50×, NA = 0.42 Mitutoyo Plan Apo NIR). The scattered light then by using a flipping mirror could be either sent to the Canon 400D CCD Camera for precise positioning of the sample or to the Horiba LabRAM spectrometer with 150 gr/mm grating to analyze the spectrum.

## Single wavelength excitation Rayleigh and Raman scattering measurements

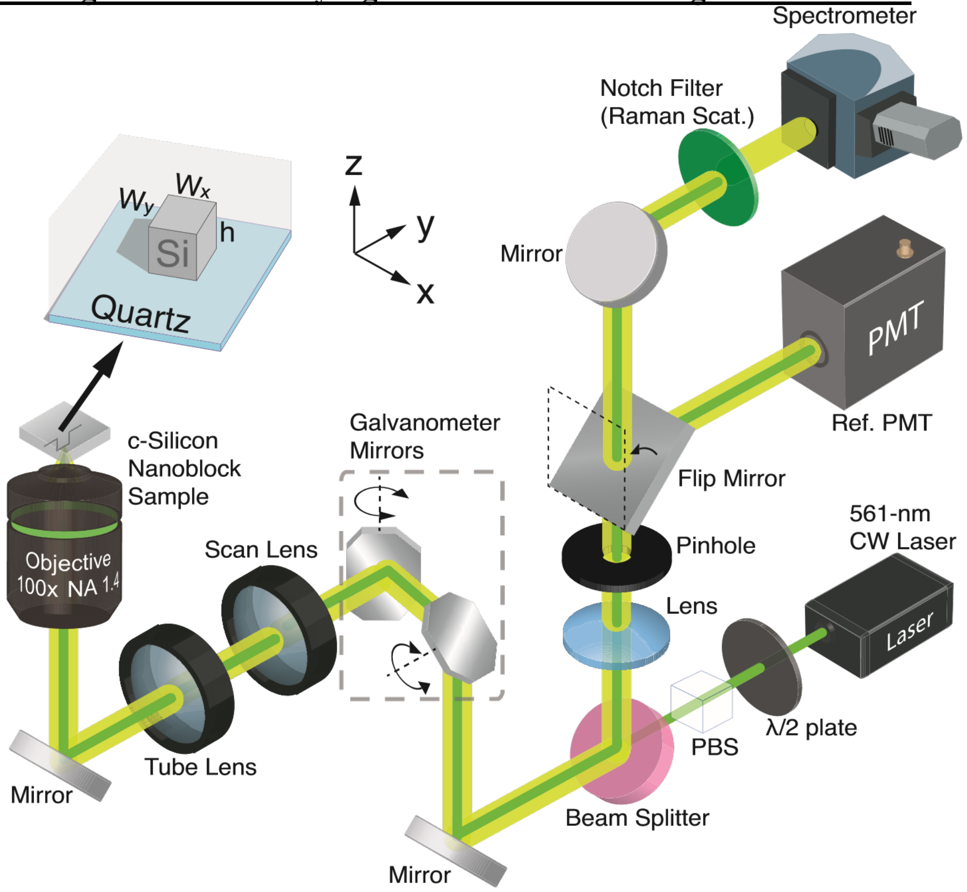


Figure S4. Laser-scanning optical setup for Raman microspectroscopy.

Figure S4 shows our experimental optical setup to obtain Rayleigh and Raman scattering with a single wavelength excitation light (Figure 3 in the main manuscript). The excitation light source is a continuous-wave laser with a wavelength of 561 nm (Jive, Cobolt, Sweden). The excitation intensity is controlled by using a half-wave plate and a polarizing beam splitter. The excitation beam is sent into a pair of galvanometer mirrors and tightly focused onto the sample by a high-NA objective (UPlanSApo 100X Oil, NA = 1.4, Olympus, Japan). By controlling the angles of galvanometer mirrors, the illumination focus spot is guided to a target silicon nanoblock. The backward scattering signals from the sample were collected by the same objective lens. Before acquiring Raman spectroscopy, we obtained a 2D scanning image with a built-in photomultiplier tube to confirm the nanoblock was at the focal position of illumination light. Then, the Raman scattering spectrum was measured by a spectrometer equipped with a cooled CCD camera (Kymera-328i, Andor, UK). A notch filter (NF561-18, Thorlabs, USA) was placed in front of the spectrometer to block the residual reflected laser and Rayleigh scattering[5,6]. During the acquisition of Raman signal, we continuously scanned the illumination focal spot onto the silicon nanoblock to avoid excess heat damage to the sample.

It also means that the detected signal consists of the sum of the scattering signals from different illumination positions, and thus no local effect due to the spatial distribution of illumination intensity is expected in the detected signal.

## • Section 4. Optical properties simulations

In this study, we used the COMSOL Multiphysics software to perform numerical simulations of Rayleigh scattering based on the finite element method. In the simulation model, we considered the geometry of a crystalline silicon nanoblock placed on a quartz substrate and immersed in an immersion oil. The refractive index of immersion oil is n = 1.515. While the refractive index value of the quartz substrate is 1.54, for the sake of simplicity and to avoid complications with reflection and transmittance, we decided to use the same refractive index value for both quartz and oil (n = 1.515), as they are very close to each other. The silicon nanoblock was illuminated with a focused beam at a wavelength of 561 nm, through an objective with a numerical aperture of 1.4, which was calculated by accurate plane-wave expansion built-in in COMSOL to model a Gaussian beam. The collected backward scattering signal was calculated by integrating the Poynting vector at the frequencies of the scattering signal over the substrate-side region of the hemisphere corresponding to the detector aperture.

The temperature-dependent refractive index values of silicon were adopted from our previous work[5]. Silicon's temperature-dependent complex refractive index values were experimentally measured up to 700 K using a commercial ellipsometer equipped with a sample heater (M-2000 DI ellipsometer, JA Woollam, NE). The refractive index values at the temperature above 700 K were obtained by fitting the following equations to the experimentally measured values.

$$n(T) = a_n(T - 300) + n_n \tag{16}$$

$$k(T) = a_k exp(\frac{T}{T_k}) \tag{17}$$

By fitting, the constants $a_n$ $n_n$ $a_k$ and $T_k$ were found to be $2.88 \times 10^{-4}$, 3.98, $1.04 \times 10^{-2}$, and 353, respectively.

## • Section 5. Intensity-dependent Raman Scattering Spectra from Bulk Silicon

Figure S5 shows the Stokes and anti-Stokes Raman scattering spectra from a bulk crystalline silicon with various excitation intensities.

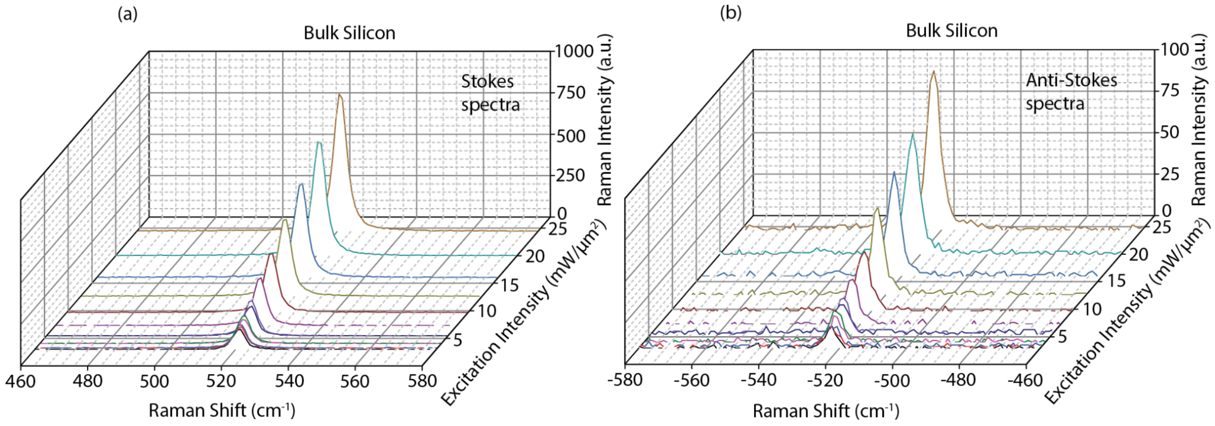


Figure S5. Raman scattering spectra of bulk silicon with various excitation intensities. (a) Stokes scattering spectra. (b) Anti-Stokes scattering spectra.

Dependencies of Stokes peak intensity, Stokes-anti-Stokes intensity ratio, and Stokes peak wavenumber on the excitation intensity are shown in Figure S6. Raman scattering intensity of bulk silicon linearly increases with the excitation intensity, unlike the silicon nanoblock. In addition, the Stokes-anti-Stokes intensity ratio and the Stokes peak position do not exhibit large changes, even though excitation intensity is increased.

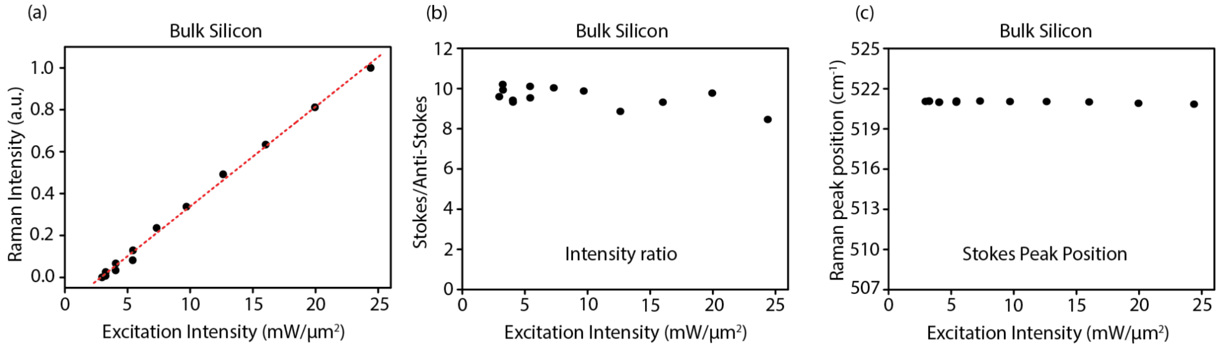


Figure S6. (a-c) Dependencies of Stokes peak intensity (a), Stokes-anti-Stokes intensity ratio (b), and Stokes peak wavenumber (c) of bulk silicon on the excitation intensity.

By using the results of Figure S6, we performed Raman thermometry of bulk silicon (see Figure S7). In both thermometry results based on the Stokes-anti-Stokes intensity ratio and peak

position shift, the estimated temperatures do not show a significant rise with the increase of the excitation intensity, confirming the fact that the photothermal effect is inefficient in increasing the temperature of bulk silicon due to its high heat capacity by its much larger volume than those of nanostructures.

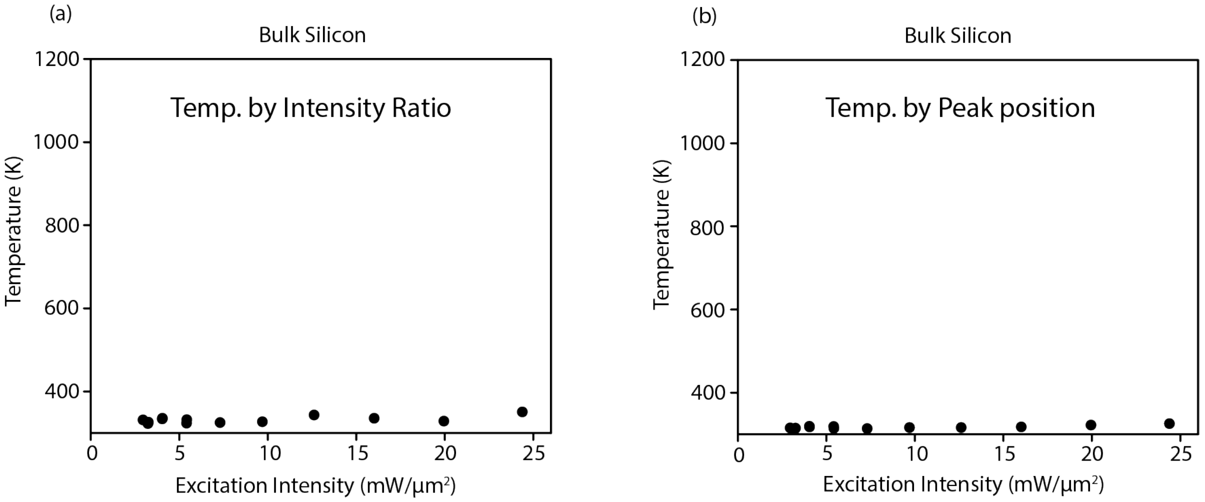


Figure S7. (a,b) Raman thermometry of bulk silicon, based on Stokes-anti-Stokes peak intensity ratio (a) and peak wavenumber shift (b).

## • Section 6. Intensity-dependent Raman Spectra from 180 nm and 250 nm Silicon Nanoblocks

Figure S8 is the Stokes and anti-Stokes Raman scattering spectra of 180 nm and 250 nm width silicon nanoblocks with various excitation intensities. The Stokes and anti-Stokes Raman scattering intensity of 180 nm silicon nanoblock shows a decrease by increasing excitation intensity, i.e., the modulation of Raman scattering intensity, due to photo-thermo-optical effects with Mie resonance. However, the 250 nm silicon nanoblock does not show significant modulation of Raman scattering intensities.

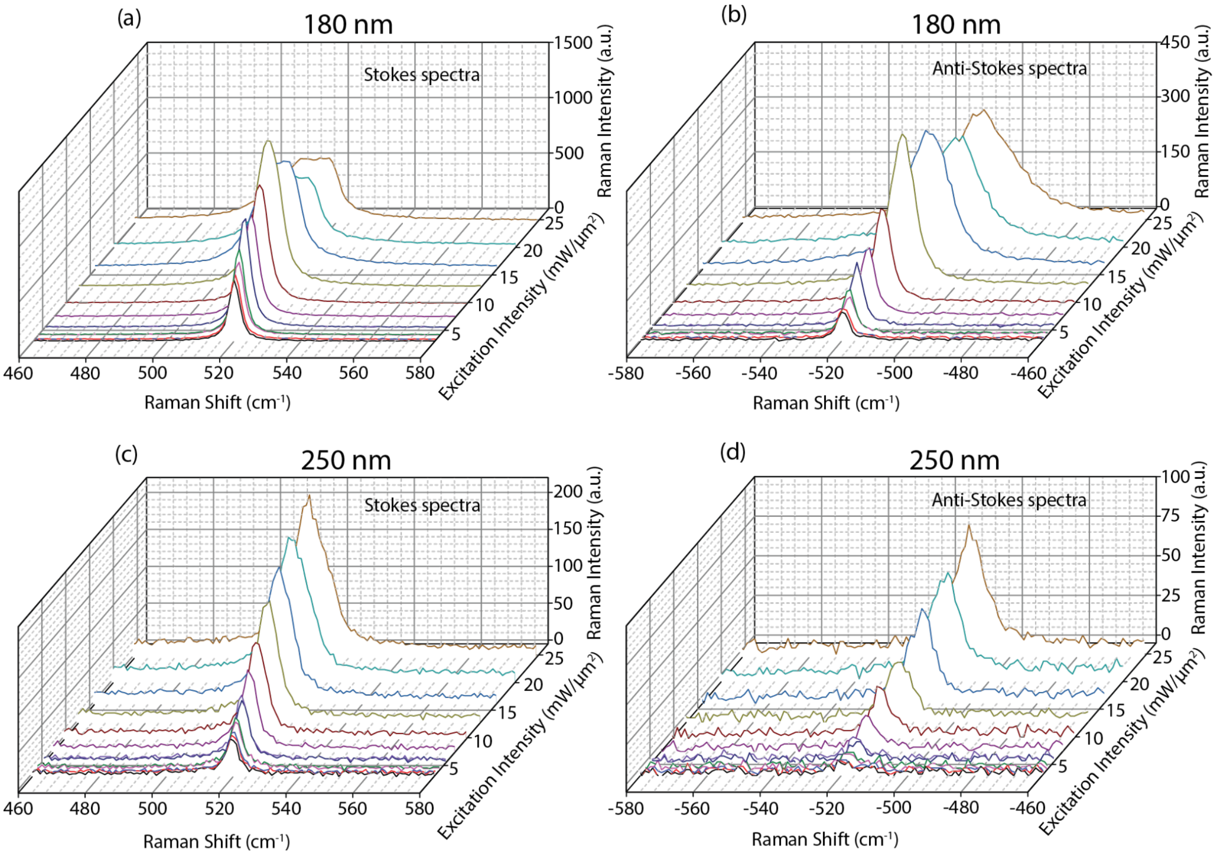


Figure S8. Raman scattering spectra of silicon nanoblocks. (a, b) Stokes (a) and Anti-Stokes (b) spectra of 180 nm side width silicon nanoblock. (c,d) Stokes (c) and anti-Stokes (d) spectra of 250 nm side width nanoblock.

Figure S9 are dependencies of the Stokes-anti-Stokes intensity ratio and Stokes peak wavenumber of silicon nanoblock on the excitation intensity, calculated by the results of Figure S8. The peak intensity and the peak position were determined by applying the Lorentzian curve fitting to the experimental Raman spectra. Different from the results of bulk silicon (Figure S6), both Stokes-anti-Stokes intensity ratio and peak position shift of silicon nanoblocks largely varies with the increase of the excitation intensity, implying the estimated temperatures exhibit

significant changes due to the photothermal effect. Based on this result, we derived the temperature of silicon nanoblocks, as shown in Figure 4 of the main manuscript.

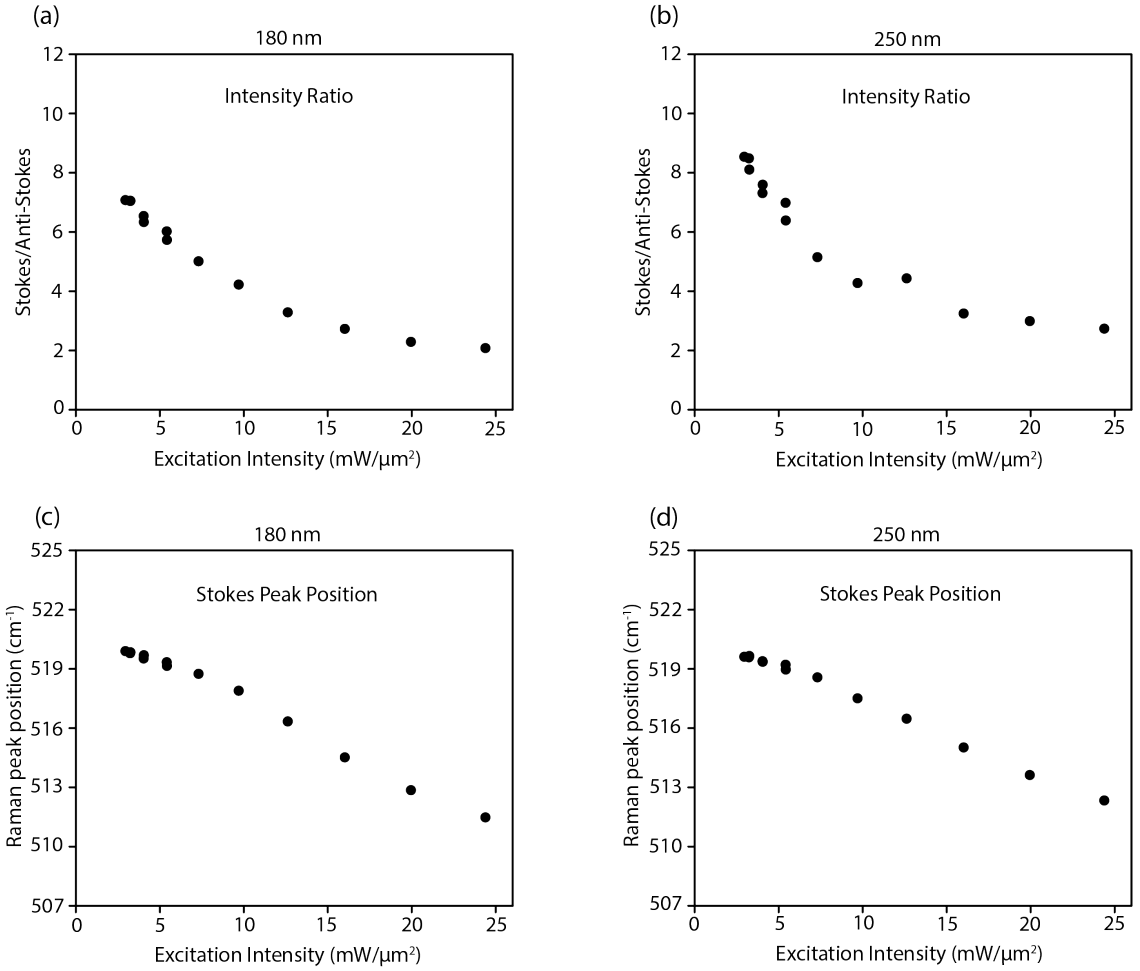


Figure S9. Dependencies of Stokes-anti-Stokes intensity ratio and Stokes peak wavenumber of silicon nanoblock on the excitation intensity. (a, b) The stokes-anti-Stokes intensity ratio of 180 nm (a) and 250 nm (b) side width nanoblock. (c, d) Stokes peak wavenumber of 180 nm (c) and 250 nm (d) side width nanoblock.

## • Section 7. Raman thermometry

In this work, we determined the temperatures of the silicon nanoblocks based on the Stokes and anti-Stokes intensity ratio, and Stokes peak wavenumber shift of the experimentally measured Raman scattering spectrum.

Stokes to anti-Stokes intensities ratio exhibits an exponential dependence on the temperature[7] because of the Boltzmann distribution of the phonon population. The relationship between the Stokes-anti-Stoke peak intensity ratio and temperature is given as follows,

$$I_S/I_{aS} = exp(\frac{\hbar\,\Omega_0}{k_B T}) \tag{18}$$

where $I_S$ and $I_{aS}$ are Stokes and anti-Stokes scattering intensities, respectively, $\hbar$ is the Dirac's constant, $\Omega_0$ is the optical phonon frequency of material that corresponds with the frequency of Raman shift, $k_B$ is the Boltzmann constant, and $T$ is the absolute temperature.

Raman peak frequency shift of an optical phonon also varies depending on temperature because the thermal expansion impacts the lattice structure, and is utilized as a parameter of Raman thermometry. The relationship between Raman shift and the temperature is shown in the equation below.[8]

$$\Omega(T) = \Omega_0 + A(1 + \frac{2}{e^x - 1}) + B(1 + \frac{3}{e^y - 1} + \frac{3}{(e^y - 1)^2}) \tag{19}$$

where $\Omega_0$ is the optical phonon frequency of material at room temperature, $A$ and $B$ are constants ($A = -2.96cm^{-1}$, and $B = -0.174cm^{-1}$), $x = \frac{\hbar\,\Omega_0}{2k_B T}$ $y = \frac{\hbar\,\Omega_0}{3k_B T}$ for crystalline silicon, $\hbar$ is the Dirac's constant, $k_B$ is the Boltzmann constant, and $T$ is the absolute temperature. We used this equation to determine the temperature from Stokes's peak wavenumber.

## • Section 8. Raman Scattering Calculation

The analytical formulations of Raman scattering versus excitation intensity, which are represented in Eq. 1 and Eq. 2 of the main manuscript, are utilized only to qualitatively explain the contribution of the Purcell effect on the Raman scattering, they were not directly utilized to quantitatively assess the experimental results. This is because the actual optical properties of the silicon nanoblock can be governed by several modes which are spectrally closed to each other, over the assumption of our analytical model (i.e. single mode approximation). Instead, we relied on the numerical calculation by using FEM software (COMSOL Multiphysics, COMSOL Inc.), which allows us to simulate the complex Raman numerical beyond single mode approximation.

So, to model the Raman response, we relate the physics of the two Electromagnetic Waves, Frequency Domain (EWFD) interfaces, corresponding to different frequencies $\omega$, $\omega_S$. The first interface involved solving the equation for the electric field amplitude $E(r,\omega)$ at the pump frequency. The second interface corresponded to the Raman frequency and was implemented by setting the induced Raman polarization at this frequency $P_R^{\sigma}(r,\omega_s) = \alpha_R \widehat{R^{\sigma}} E^{\sigma}(r,\omega)$, where $\alpha_R$ is the phonon-polarization independent scalar, $E^{\sigma}(r,\omega)$ is the component ($\sigma = x,y,z$) of the electric field inside the nanoparticle and $\widehat{R^{\sigma}}$ is the following known form of the Raman tensor in silicon.

$$R^x = \begin{pmatrix} 0 & 0 & 0 \\ 0 & 0 & 1 \\ 0 & 1 & 0 \end{pmatrix},\ R^y = \begin{pmatrix} 0 & 0 & 1 \\ 0 & 0 & 0 \\ 1 & 0 & 0 \end{pmatrix},\ R^z = \begin{pmatrix} 0 & 1 & 0 \\ 1 & 0 & 0 \\ 0 & 0 & 0 \end{pmatrix}$$

To take into account the spatial incoherence of Raman radiation, we independently set the induced polarization in different subdomains of the structure, namely, identical subcubes constituting the original cubic nanoparticle. As a result, the total Raman response from the entire nanocube was determined by summing the independent responses from each subdomain. We ensured that dividing the particle into 27 subdomains was sufficient to describe the incoherent response, since further division (for example, into 64 subdomains) did not lead to a change in the intensity dependence of the Raman response signal.

The above text describes a computer simulation that considers the influence of Purcell effect on the Raman scattering dependence on excitation intensity. To neglect the Purcell effect, one can replace the complex refractive index of the particle material with the corresponding

values for air in the second EWFD interface, which is responsible for solving Maxwell's equations at the Raman frequency.

Figure S9(a) and S9(b) respectively show the Raman scattering intensity for 180 nm and 250 nm silicon nanoblocks. The numerical calculation results predict that the nonlinearities of Raman intensities from both W = 180 nm and W = 250 nm silicon nanoblocks. In addition, the result also indicates W = 250 nm silicon nanoblock shows a larger difference in Raman scattering intensity between with and without considering the Purcell effect, which confirms the more significant contribution of the Purcell effect than that of W = 180 nm nanoblock. Nevertheless, the correspondence of the simulated Raman scattering dependency with the experimental results (Figure 3(e) and 3(f) in the main manuscript) is still not very good in W = 250 nm silicon nanoblock. The improvement of the numerical simulation algorithm, accounting for temperature-dependent optical material properties, as well as for the incoherent nature of the Raman emission, would be our future work.

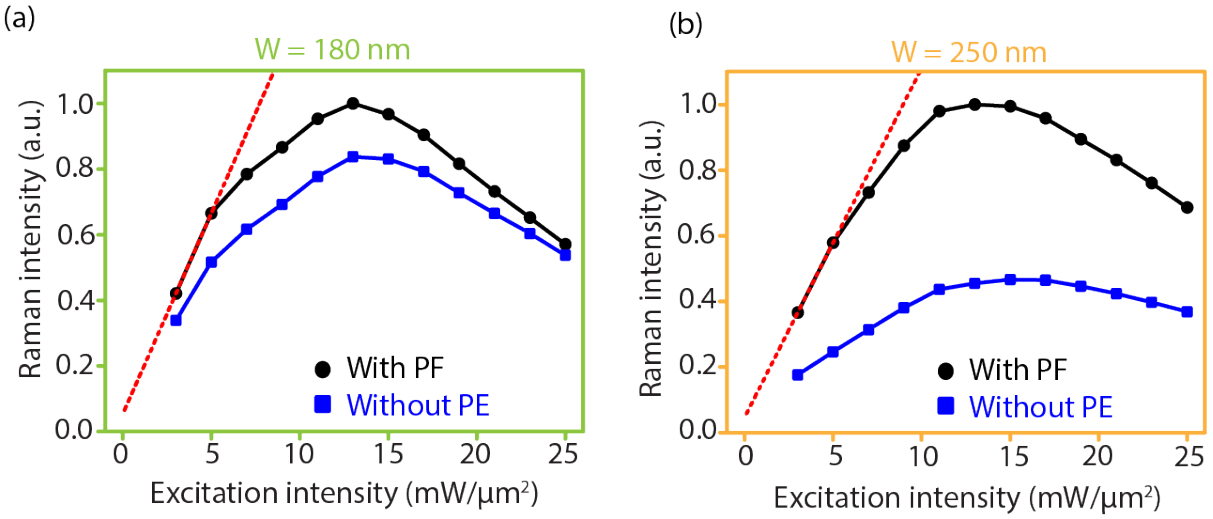


Figure S10: Numerically calculated relationships between excitation and Stokes Raman scattering intensity from W =180 nm (a) and W = 250 nm (b). Black and blue lines respectively, indicate the graph with and without considering the Purcell effect (PE).

## References


[1] H. A. Haus, *Waves and Fields in Optoelectronics*, Prentice Hall, **1984**.
[2] M. B. Doost, W. Langbein, E. A. Muljarov, *Phys. Rev. A* **2014**, *90*, 013834.
[3] K. Frizyuk, M. Hasan, A. Krasnok, A. Alú, M. Petrov, *Phys. Rev. B Condens. Matter* **2018**, *97*, 085414.
[4] T. Nagata, H. Kanemaru, M. Ikegami, Y. Nagatomo, R. Nakamura, M. Handa, K. Uchibori, in *2011 International Electron Devices Meeting*, IEEE, **2011**.
[5] Y.-S. Duh, Y. Nagasaki, Y.-L. Tang, P.-H. Wu, H.-Y. Cheng, T.-H. Yen, H.-X. Ding, K. Nishida, I. Hotta, J.-H. Yang, Y.-P. Lo, K.-P. Chen, K. Fujita, C.-W. Chang, K.-H. Lin, J. Takahara, S.-W. Chu, *Nat. Commun.* **2020**, *11*, 4101.
[6] C.-H. Li, Y.-L. Tang, J. Takahara, S.-W. Chu, *J. Chem. Phys.* **2021**, *155*, 204202.
[7] T. Zhang, Y. Che, K. Chen, J. Xu, Y. Xu, T. Wen, G. Lu, X. Liu, B. Wang, X. Xu, Y.-S. Duh, Y.-L. Tang, J. Han, Y. Cao, B.-O. Guan, S.-W. Chu, X. Li, *Nat. Commun.* **2020**, *11*, 1.
[8] M. Balkanski, R. F. Wallis, E. Haro, *Phys. Rev. B Condens. Matter* **1983**, *28*, 1928.
